# Applications of Pose Estimation in Human Health and Performance across the Lifespan

**DOI:** 10.3390/s21217315

**Published:** 2021-11-03

**Authors:** Jan Stenum, Kendra M. Cherry-Allen, Connor O. Pyles, Rachel D. Reetzke, Michael F. Vignos, Ryan T. Roemmich

**Affiliations:** 1 Center for Movement Studies, Kennedy Krieger Institute, Baltimore, MD 21205, USA; jstenum1@jhmi.edu; 2 Department of Physical Medicine and Rehabilitation, Johns Hopkins University School of Medicine, Baltimore, MD 21205, USA; kcherry6@jhu.edu; 3 Johns Hopkins Applied Physics Laboratory, Laurel, MD 20723, USA; Connor.Pyles@jhuapl.edu (C.O.P.); Mike.Vignos@jhuapl.edu (M.F.V.); 4 Center for Autism and Related Disorders, Kennedy Krieger Institute, Baltimore, MD 21211, USA; Reetzke@kennedykrieger.org; 5 Department of Psychiatry and Behavioral Sciences, Johns Hopkins University School of Medicine, Baltimore, MD 21205, USA

**Keywords:** pose estimation, movement tracking, computer vision, artificial intelligence, markerless motion capture, assessment, kinematics, development, machine learning

## Abstract

The emergence of pose estimation algorithms represents a potential paradigm shift in the study and assessment of human movement. Human pose estimation algorithms leverage advances in computer vision to track human movement automatically from simple videos recorded using common household devices with relatively low-cost cameras (e.g., smartphones, tablets, laptop computers). In our view, these technologies offer clear and exciting potential to make measurement of human movement substantially more accessible; for example, a clinician could perform a quantitative motor assessment directly in a patient’s home, a researcher without access to expensive motion capture equipment could analyze movement kinematics using a smartphone video, and a coach could evaluate player performance with video recordings directly from the field. In this review, we combine expertise and perspectives from physical therapy, speech-language pathology, movement science, and engineering to provide insight into applications of pose estimation in human health and performance. We focus specifically on applications in areas of human development, performance optimization, injury prevention, and motor assessment of persons with neurologic damage or disease. We review relevant literature, share interdisciplinary viewpoints on future applications of these technologies to improve human health and performance, and discuss perceived limitations.

## 1. Introduction

Humans have long been interested in quantitative measurement of our movements [1,2]. This is evident in many aspects of life: an Olympic judge scrutinizes and scores a figure skater’s performance; a physical therapist measures a patient’s walking speed to assess mobility; a running coach inspects and adjusts a distance runner’s foot-strike pattern to prevent injury. We also interpret the movements of others to communicate (e.g., sign language) or make inferences about emotional state (i.e., “reading body language”; [3,4,5]).

**Figure 1:**
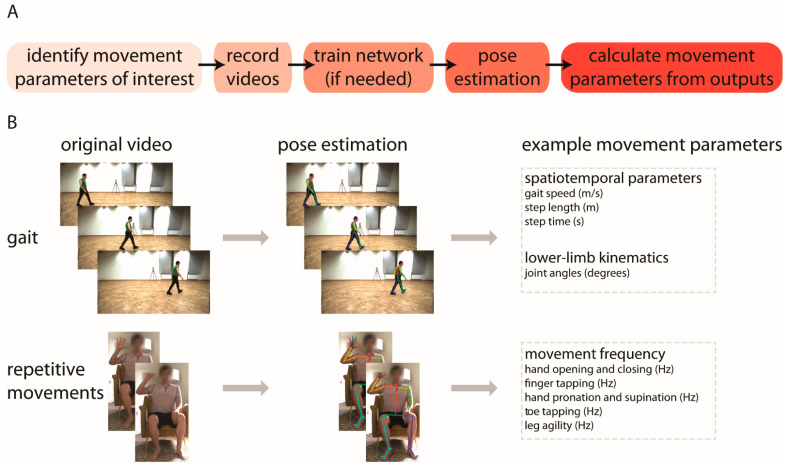
(**A**) Basic workflow for using pose estimation to measure movement kinematics from video; (**B**) Example applications of using pose estimation to quantify spatiotemporal and kinematic gait parameters (top) and frequencies of repetitive upper and lower extremity movements (bottom). These applications are described in greater detail in [14,15]. The gait images shown in (**B**) are taken from the GPJATK dataset [16].

In this review, we focus on applications of human pose estimation, an emerging technology for quantitative measurement of human movement kinematics [6,7,8,9,10,11,12,13]. Pose estimation algorithms use computer vision to identify key landmarks on the body (e.g., fingertip, elbow, knee) from simple digital videos that can be recorded using common household devices (example workflow and applications are shown in Figure 1A,B, respectively). This simplicity offers exciting potential for measuring whole-body kinematics in nearly any setting, with minimal costs of money, time, and effort. We also see significant opportunities for the ongoing maturation and validation of these approaches to offer robust supplements or alternatives to subjective visual motor assessments and to improve accessibility to measurement of movement kinematics by removing long-standing barriers. The ability to capture quantitative, whole-body kinematics using a household device could substantially reduce reliance on traditional methods that are inaccessible or data-limited, such as expensive research-grade motion capture systems or wearable devices.

We focus specifically on applications of human pose estimation for improving human health and performance. We note that pose estimation algorithms are used for many other applications (e.g., intelligent video surveillance [17], activity recognition [18], sign language translation [19]), and prior reviews have discussed technical aspects of various algorithms and their perceived advantages and disadvantages [20,21,22]. Here, we focus less on the technical aspects of pose estimation and instead discuss applications of these algorithms, both in terms of current applications and those that we perceive may be possible in the future. We cover areas of application across the human lifespan, including human development, human performance optimization, musculoskeletal injury prevention, and motor assessment of persons with neurologic damage or disease.

We also integrate the clinical perspective on pose estimation applications. Much prior work on human pose estimation (including our own) has suggested promise for clinical application. However, in our view, the clinician’s (i.e., end user) viewpoint on these potential applications has not received adequate consideration or representation, and applications of pose estimation have not been contextualized within current models of clinical care. We aim to address these issues by providing an interdisciplinary perspective that integrates views from physical therapy, speech-language pathology, movement science, and engineering.

## 2. What Is Pose Estimation?

Markerless human pose estimation relies on recent advances in computer vision to automatically track anatomical landmarks—so-called keypoints—of the human body from digital videos. Examples of possible tracked keypoints include the ankle, knee, hip, wrist, elbow, shoulder, foot (e.g., heel, big toe, and small toe), hand (e.g., tip and three joints of every finger), and face (e.g., ears, eyes, nose, and mouth). Current state-of-the-art algorithms used to track human poses have been trained on large datasets of digital images and/or videos of human movement in which keypoints have been manually annotated [23,24]. The trained algorithms can then track new, unlabeled videos of humans. This enables automated, video-based human movement tracking, with the greatest accuracy achieved for movements similar to those in the training dataset.

The primary output from pose estimation is a series of two-dimensional pixel coordinates of the tracked keypoints, as they appear projected onto the image sensor of the camera. From the two-dimensional pixel coordinates, different approaches of analyzing and processing data have been reported, and fall into three broad categories. First, some studies use the output to represent planar two-dimensional kinematics of human movement, from which specific metrics of interest can be calculated [15,25,26,27,28]. An example of an instance in which this approach may be appropriate is capturing a video of the sagittal view of human locomotion and subsequently calculating sagittal gait kinematics (e.g., lower limb joint angles). Second, it is possible to reconstruct three-dimensional kinematics of human movement if capturing videos from multiple viewpoints using at least two cameras [29,30,31]. This approach offers significant advantages over a single camera view, in part because occlusions occur and out-of-plane motions are not well-captured by a single camera; however, this approach also has potential drawbacks associated with setup and computational complexity. Last, it is also possible to use the pose estimation output as an input for further processing by neural networks designed to predict specific metrics of interest [32,33,34]. Subsequent processing by neural networks may be appropriate when predicting a scalar value such as peak knee flexion during walking or clinical ratings, but this approach may be less accurate when predicting frame-by-frame time-series data. This inaccuracy is commonly due to the fact that most algorithms do not aim to minimize frame-to-frame variation when performing pose estimation with video data.

These diverse approaches to data analysis of pose estimation of human movement make it possible to obtain many parameters associated with movement. For example, pose estimation has been used to study human locomotion [15,34,35] and provide kinematic measures such as lower limb joint angles; spatiotemporal measures such as gait speed, step length, and step time; and clinical ratings such as the Gait Deviation Index in patients with cerebral palsy or MDS–UPDRS gait scores for persons with Parkinson’s disease. Other studies have used pose estimation to assess neuromotor risk and development in human infants [36,37]. These areas of application are introduced briefly here, but will be covered in greater detail in later sections of this manuscript.

## 3. What Tools Are Available?

Several different algorithms for pose estimation have been published over the past decade (e.g., OpenPose [13], DeepLabCut [12], DeepPose [10], DeeperCut [8], AlphaPose [38], ArtTrack [7]). Using these algorithms, it is possible to take advantage of pretrained networks that are freely available, or train new networks customized for various research or clinical needs. For example, a commonly used pretrained network is the human pretrained demo of OpenPose that includes keypoints of the body, feet, hands, and face [13,39] and has been used in several recent studies for quantitative analysis of human movement [15,26,29,31,34,40].

The computations needed for training a new network and tracking new videos often require intensive computing capabilities. Therefore, the computing power of a graphics processing unit (GPU) may be necessary in order for processing times to reach acceptable limits (many algorithms provide documentation with hardware recommendations, as in [11]). If a user does not have their own GPU, some computing environments (e.g., Google Colaboratory) provide GPU access for faster processing; however, these may not be suitable for applications involving protected health information because the processing occurs externally. Processing without a GPU is slower but may be sufficient depending on the user’s time constraints and processing needs (e.g., length of videos, number of people tracked, number of keypoints tracked). Furthermore, it is also possible to use pose estimation for real-time movement tracking (as is available with OpenPose, for example [39]). This capability may be particularly useful to some users, as it could be implemented to provide real-time biofeedback for various applications. Beyond these increasingly popular deep learning approaches, other approaches also use optimization [41,42,43] and filtering [44,45] techniques to perform pose estimation.

## 4. How Can These Tools Be Used to Improve Human Health and Performance?

**Figure 2:**
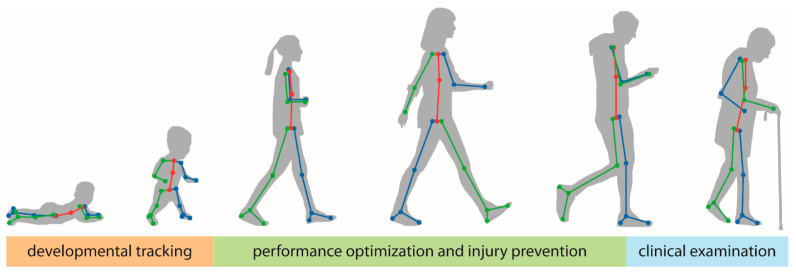
In this manuscript, we focus on three general areas of applications of pose estimation in human health and performance across the lifespan: tracking of motor and non-motor development in young children (orange), performance optimization and injury prevention in athletes and other populations that are primarily young or middle-aged adults (green), and clinical examinations of persons with neurologic damage or disease who are primarily older adults (blue).

In the following subsections, we will focus on three specific areas of application across the human lifespan: (1) human development, (2) performance optimization and injury prevention, and (3) motor assessment of persons with neurologic damage or disease (Figure 2). Certainly, many additional areas of application exist beyond the scope of this review. We focus on these applications due to the emerging nature of the relevant literature and the expertise of the authors. We expect that many of the principles discussed below are likely to generalize to other applications and/or populations of interest.

### 4.1. Tracking General Motor Development

Developmental scientists study the emergence of specific behaviors from infancy to adolescence in many different settings, including the laboratory, home environment, clinic, and classroom. Accordingly, video recordings are an integral component of most, if not all, developmental research programs. Video-based approaches have been used to study multiple domains of development, including gross and fine motor development as well as social, language, and play development [46,47,48,49]. One major limitation of current video-based approaches is the time-intensive but necessary process of manually coding child behaviors of interest by clinicians and researchers. Pose estimation technologies offer a much-needed opportunity to accelerate video coding to capture specific behaviors of interest in such developmental investigations. Due to the extensive manual video coding that has been done in the field over decades, there are large existing video databases that have already undergone human coding/reliability checks and can provide a valuable source of ground truth data for training and validation of machine learning models of development (e.g., [50]). Such approaches could further help decrease reliance on assessment tools that require the expertise and time of trained clinicians for interpretation and, in turn, offer cost-effective and scalable alternatives to more subjective measures of typical and atypical development.

**Figure 3:**
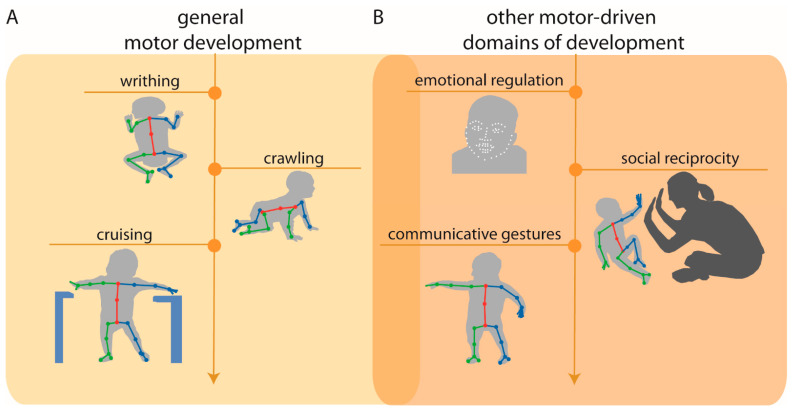
(**A**) Example applications of pose estimation to quantify early motor developmental milestones (left), including writhing movements (e.g., [51]), crawling, and cruising (e.g., [36]); and (**B**) other motor-driven domains of development, including emotional regulation, social reciprocity, and communicative gestures. Overlap between (**A**,**B**) denotes that these areas of development are intimately linked with one another. Arrow indicates that application of pose estimation is not restricted to these examples and can be applied to quantify later motor and motor-driven developmental milestones.

Although in the early stages of application, pose estimation approaches are beginning to be applied to the study of general motor development [36,51] (Figure 3A). For example, pose estimation has been used to detect normal writhing movements (i.e., typical spontaneous movements produced by newborns) vs. abnormal movements from video recordings of newborns in their first days of life [51]. Preliminary findings are promising and suggest that normal vs. abnormal writhing movements can be automatically classified with 80% accuracy, a percentage comparable to expert human classification.

As infants progress in their gross motor development, the onsets of crawling and walking—gross motor advances that allow infants to explore and learn from their environment—have been found to be intimately linked with growth in other developmental domains [52,53]. Indeed, findings from developmental science literature suggest that delays in the onset of walking may result in limited opportunities for exploration and input from caregivers and family members, leading to subsequent delays in language and social communication development [48,54,55]. As a result, it is critical to improve the early detection of delays in locomotor development in order to intervene prior to any cascading effects on other domains of development.

Researchers have begun to implement pose estimation as a useful tool for quantitative tracking of infant locomotor development. For example, Ossmy and Adolph [36] used a combination of pose estimation, machine learning, and time-series analyses to examine the role of experience in infant acquisition of interlimb coordination based on video recordings of the infants “cruising” (i.e., side-stepping with support of the upper extremities)—which is the transitional behavior between crawling and walking—at 11 months of age. More specifically, the authors used pose estimation to track frame-by-frame body movements and subsequently calculated the distance between the limbs (i.e., the distance between the hands and the distance between the feet) for each tracked video frame to extract the coordination pattern for cruising. The results of this study provided insight into the mechanisms by which infants learn to optimally cruise and, as a result, may hold implications for future work aiming to investigate early detection and intervention for delays in locomotor development.

### 4.2. Clinical Use in Pediatric Populations

Early detection of atypical development is critical for the diagnosis of congenital movement-based disorders (e.g., cerebral palsy) and neurodevelopmental disorders (e.g., autism spectrum disorder) to ensure timely access to early intervention services to improve motor outcomes (e.g., coordination, postural support) and other domains of development (e.g., social, language). Advances in pose estimation approaches and the emergence of novel machine learning-based models offer exciting potential for the assessment of movement-based predictors of clinical disorders. For example, pose estimation is beginning to be applied, not only to measure predictors of later motor-based disorders, but also predictors of other motor-driven domains of development (social communication; Figure 3B). In this subsection, we provide examples of these advances.

Cerebral palsy (CP) is the most common movement disorder in childhood, caused by abnormal neural development or injury that impairs the ability to control movement and posture [56]. Diagnosis of CP using conventional assessments typically occurs between age 12 and 24 months; however, using a combination of standardized assessments and neonatal magnetic resonance imaging (MRI), CP can be accurately predicted before 6 months corrected age [57]. Yet, there remain significant drawbacks to this approach: standardized assessments are based on subjective human observation that requires substantial training and clinical expertise, and neonatal MRI is expensive and often inaccessible in low-resource areas [58].

Recent research efforts have attempted to address these shortcomings by aiming to use video recordings to implement low-cost, automatic, objective alternatives for the detection of CP risk. Such investigations have succeeded in predicting CP based on automatic movement assessment from infant video recordings with performance comparable to standardized CP risk measures [59,60,61]. For example, in a multi-site cohort investigation, an automated, objective, movement assessment of infant video recordings was compared to standard risk assessment measures (i.e., the General Movement Assessment and neonatal neuroimaging) at 9–15 weeks corrected age to predict CP status and motor function at approximately 3.7 years of age. The results of this investigation found that the automated, video-based approach exhibited sensitivity and specificity comparable to standard measures used to predict CP [61].

There are also clear applications for pose estimation to potentially improve the early identification of neurodevelopmental disorders, such as the early detection of autism spectrum disorder (ASD). Although parents often report first concerns about ASD when their child is between 12 to 14 months of age [62,63] and reliable ASD diagnosis is possible by age 2, the majority of children with ASD remain undiagnosed until 4 years of age [64]. Shortages of ASD expert clinicians and limited capacities at autism tertiary diagnostic centers contribute to the long wait times for families [65]. Families living in rural and low-resource communities are often required to travel long distances to receive diagnostic services, placing them at an even greater disadvantage in accessing services. Indeed, a recent report indicates that approximately 84% (2635/3142) of U.S. counties do not have the necessary ASD diagnostic resources [66]. Given these barriers to a timely diagnosis, a significant portion of children with ASD are missing a critical window for early intervention services, as evidence shows that intervention before the age of 2 significantly improves behavioral and developmental outcomes for children with ASD [67,68,69]. The detrimental impact of diagnostic delays has resulted in federal prioritization of early identification of ASD and an urgency to develop accessible and accurate early screening methods [64].

Leveraging advances in machine learning, efforts have been made to develop scalable, video-based ASD screeners to improve access to diagnostic and early intervention services. For example, Crippa et al. developed an algorithm to examine the predictive value of motor behavioral biomarker measures in ASD to discriminate preschool children with ASD from children with typical development using a simple upper-limb reach-to-drop task [70]. The resulting model showed an accuracy rate of 96.7%, suggesting that video-based approaches combined with machine learning can be a useful method of classification and discrimination in the diagnostic process [70].

The emerging evidence supporting the application of automated, video-based assessments to monitor general gross motor development and promote early detection of both motor-based and neurodevelopmental disorders is promising. In order to establish the clinical utility of pose estimation, future work is needed to examine the feasibility and acceptability of clinician use of such techniques.

### 4.3. Human Performance Optimization, Injury Prevention, and Safety

Numerous applications of pose estimation exist within optimization of human performance and safety, with these applications spanning injury risk assessment, rehabilitation, and enhancing human performance. This application space commonly consists of some type of instructor, such as a coach, trainer, or clinician, attempting to assess an individual’s movement patterns to determine whether the individual is at an increased risk for injury, is moving differently from a healthy, uninjured individual, or is moving with some level of inefficiency that can be modified to improve performance. Within injury assessment, common applications of pose estimation have been to evaluate an individual’s risk for specific musculoskeletal injuries and to perform a post-hoc analysis following the occurrence of an injury. For example, two-dimensional pose estimation techniques have been applied to develop proof-of-concept screening technologies that detect abnormal gait patterns during walking and running [71,72,73,74,75], fall detection [76,77,78], abnormal movements that are indicative of injury risk in manual labor work environments [79,80,81], and risk of sports-related injury, such as anterior cruciate ligament rupture [82,83,84]. Post-hoc analysis following an injury has primarily been targeted towards sports performance applications and focused on understanding mechanisms of injury, with the ultimate goal of developing techniques to mitigate injury risk [85,86].

Applications of pose estimation to rehabilitation following injury or surgery typically focus on using these techniques to monitor an individual’s return to normal movement patterns and to guide the motion of rehabilitation technology that is designed to interface with a patient. Pose estimation techniques have been used to measure a patient’s range of motion and movement during functional exercises and assess their progression towards a healthy range of motion [87,88,89]. In particular, there has been an emphasis on the use of pose estimation to monitor rehabilitation progress outside of the clinic, such as in home or on an athletic field [90,91,92,93]. Additionally, many technologies have been designed to actively interface with an individual to either support their movement during rehabilitation or to help provide a mechanical stimulus to enhance rehabilitation. These technologies are commonly referred to as rehabilitation robotics, and techniques have been developed that leverage pose estimation to inform the movement of these systems [94,95,96,97].

The use of pose estimation for enhancing human performance remains a challenging application, given the large range of joint articulation, out of plane motion, and fast movements that can be difficult to capture with the relatively slow sampling rates of common video recording devices and risk of occlusion that occurs in these applications [98,99]. However, a number of proof-of-concept systems have been developed to inform pose of an athlete during training, particularly for sports in which success for the athlete is directly linked to pose (e.g., gymnastics and skiing) [100,101,102]. Development of new pose estimation techniques for human performance applications have focused on achieving high accuracy with ‘in the wild’ pose estimations, given the importance of performing these measurements outside of the lab in these applications [11,103,104]. While this previous research has demonstrated applications that may be made possible with pose estimation, very few of these proof-of-concept technologies have made the transition to regular use in a clinical, athletic, or other relevant environments. This likely derives from the fact that many unique requirements arise when attempting to apply these techniques to human performance applications outside of the laboratory.

For pose estimation to influence the broader human performance community, including non-clinical populations, research must drive towards robust ‘in the wild’ pose estimation encompassing a range of environments and populations. To this end, we will define desirable components of an ideal dataset for pose estimation algorithm development, training, and validation. Future studies should focus on capturing and making available these datasets to expand the application space of pose estimation or define functional limitations of the current hardware or software technology.

Many injury and performance evaluations are based on highly dynamic motion analysis [85,86,105], requiring that any pose estimation validation datasets should include accurate ground truth measurements of human joint kinematics for as many degrees of freedom as feasible. Ideally, this will include kinematics of complex joints, such as the ankle, wrists, intervertebral joints, and scapular motion—all of which play a key role in many injuries and are not estimated in most existing pose estimation techniques. Linear kinematics of the various body components should also be reported on, especially in relation to conditions that result from impact injuries (e.g., traumatic brain injury, chronic traumatic encephalopathy) [106]. Optical motion tracking is currently the gold standard for such ground truth measurements, but further accuracy (and cost) improvements are desirable due to artifacts arising from relative marker motion with respect to the underlying bony anatomy [107]. Therefore, researchers should aim to account for these artifacts within the pose estimation process.

Validation datasets should be captured outside of laboratory environments and include complexities such as partial occlusion (self-occlusion, inter-subject occlusion, environmental occlusion), various illuminations, loose-fitting clothing, and multiple camera standoffs or viewing angles. Recent examples of pose estimation outside of the lab are primarily based on monocular RGB images [108,109,110,111]. However, these techniques are generally less accurate—especially in three dimensions—when compared to laboratory pose estimation. The fusion of other pose estimation modalities, including inertial measurement units and infrared imaging, with single or multi-view RGB images is a promising direction for improved pose estimation [112], and should be included in validation datasets, such as those provided by Malleson et al. [113].

As new pose estimation algorithms are developed for human performance applications, special consideration should be given to the evaluation metrics reported. Motion type classification is of limited usefulness for in-depth biomechanical analysis and, instead, joint kinematic errors should be reported for each degree of freedom. Furthermore, estimation accuracies should be reported under varying conditions, including differences between lab-based and outdoor estimations. Finally, the computational cost per frame of pose estimation should be reported to understand applicability to real-time, highly dynamic application spaces [113].

### 4.4. Clinical Motor Assessment in Adult Neurologic Conditions

Clinical assessments and the resulting outcome measures are critical to motor rehabilitation in adults with neurologic conditions. These clinical assessments are typically administered to capture either a patient’s status at a specific point in time or to track their motor function longitudinally. When administered at a single time point, assessments are used to classify the severity of an individual’s deficits. When administered longitudinally, assessments are commonly used to track disease progression/regression, measure recovery, or evaluate the effectiveness of an intervention.

The International Classification of Functioning, Disability and Health (ICF) is a common, widely accepted framework developed by the World Health Organization for describing health and disability at individual and population levels [114]. It provides standard language and has a wide range of uses across different sectors by identifying three primary levels of human functioning:

1. *Body structures and functions* are anatomical parts of the body and physiological functions of the body systems, respectively. The term impairment refers to problems in *body structure or function*.
2. *Activity* is the execution of a task or action by an individual. The term activity limitation describes difficulties with completion of an *activity*.
3. *Participation* is involvement in a life situation. Participation restrictions are problems that an individual encounters during *participation* in real-world situations.

To provide a concrete example of how this framework is used, consider a person who has experienced a stroke. This person might experience changes in all three levels of human functioning: the impairment of left-sided hemiparesis (*body structures and functions* level), the activity limitation of difficulty walking (*activity* level), and the participation restriction of inability to attend their desired religious activities (*participation* level). One can quickly observe that, while the three levels may be related to one another, there are independent needs for quantitative measurement within each level. In other words, there are needs for quantitative measurement of the hemiparesis, daily walking activity, and the inability to attend religious activities in this particular example.

Clinical outcome measures for each level of the ICF are administered as a part of routine clinical practice. Current measures of impairment involve a skilled clinician observing a patient as they perform a series of movements designed to expose deficits in *body structure and function*. For instance, one item on the Fugl–Meyer Assessment—a widely used quantitative measure of motor impairment after stroke—involves asking the patient to move their hand from the contralateral knee to ipsilateral ear while individual elements (e.g., shoulder retraction, shoulder elevation, elbow flexion, forearm supination) of this movement are scored subjectively from 0 to 2 [115]. Measures of activity limitations involve the patient performing one or more tasks that simulate *activities* encountered in daily life. An example of an ecologically valid task is the water pouring item of the Action Research Arm Test—an extensively used activity level measure for people with stroke [116]—where the person pours water from one glass to another. Lastly are measures of participation restrictions, which are often self-reported measures of the person’s perceptions of their movement abilities and resulting impact on their quality of life (e.g., the Stroke Impact Scale [117], a self-report questionnaire that evaluates disability and health-related quality of life after stroke) and daily *participation*. The data gathered from existing outcome measures are valuable for their use in diagnosing movement disorders, establish rehabilitation goals, and track changes in patient status.

**Figure 4:**
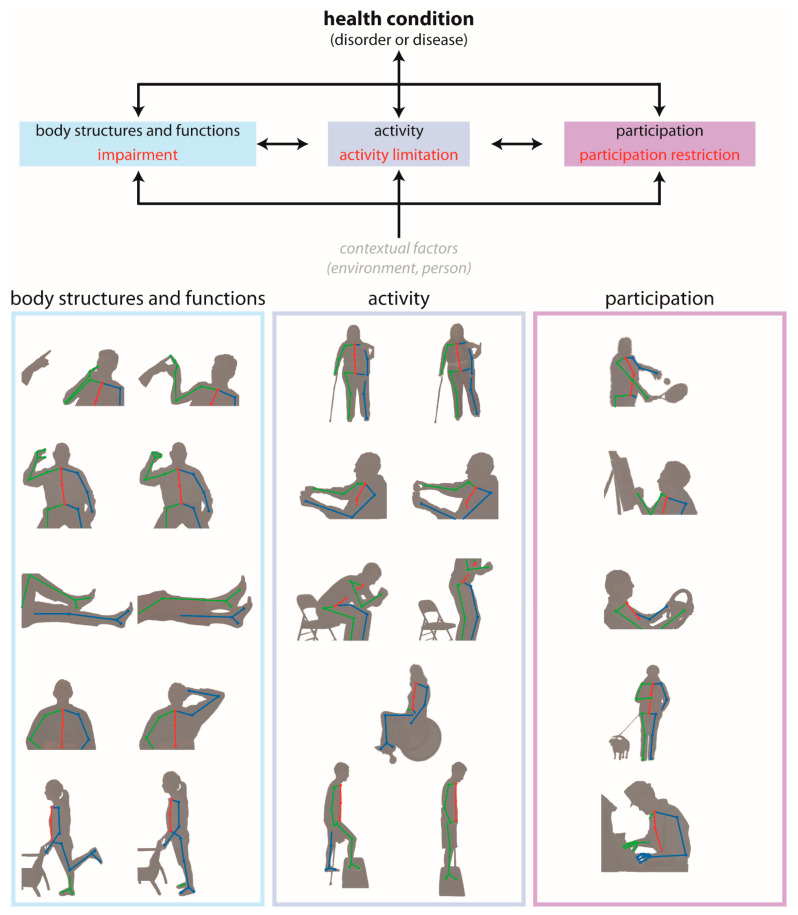
Depiction of potential applications of pose estimation for movement tracking during clinical assessments across the domains of the International Classification of Functioning, Disability and Health (ICF) model. For instance, finger–nose coordination testing the body structures and functions domain (**left**), walking assessment in the activity domain (**middle**), and playing tennis in the participation domain (**right**).

Pose estimation tools have the potential to address two important challenges that exist within current clinical assessments spanning all three levels of the ICF (Figure 4). First, they can increase the accuracy, precision, and frequency with which movement kinematics are measured and assessed. Presently, *body structure/function* and *activity* level assessments primarily rely on visual observation of movement or task performance, and many are scored on ordinal scales that require a clinic visit or other similarly time-consuming interaction for both patients and their providers. Pose estimation offers the potential to provide precise, quantitative, and continuous data about single joint or whole-body movements through short video recordings that could be recorded in virtually any setting with much higher frequency. This opportunity to obtain frequent, quantitative motor assessments could significantly enhance the abilities of clinicians to detect and track impairments and activity limitations in their patients longitudinally. Second, current assessments of participation restrictions are almost exclusively self-reported. The self-report format has been necessary due to the difficulty of measuring movement kinematics in the home, but many self-report measures lack reliability and often do not correlate with clinically-administered motor assessments. There is clear potential for the propagation of telerehabilitation and pose estimation tools to make a significant impact in this area by providing significantly improved accessibility for clinicians and researchers to obtain quantitative data about how people move and participate in their home and community environments.

The uses of pose estimation in clinical populations are expanding, but ultimately remain in the beginning stages. At the *body structure/function* level, early work has involved detecting hallmark motor signs in persons with Parkinson’s disease (PD). For instance, dyskinesia is an involuntary movement of the head, arm, leg, or entire body. Dyskinesia is commonly seen in persons with PD, often as a side effect of long-term levodopa treatment. A number of recent studies have used pose estimation to assess dyskinesias in persons with PD and found similar or superior performance with standard clinical assessments [118,119,120]. Bradykinesia, or slowness of movement, is another cardinal motor sign of PD. Liu et al. report that their computer vision-based method was 89.7% accurate in quantifying bradykinesia severity in people with PD as they performed repetitive movements including finger tapping, hand clasping, and alternating hand pronation/supination movements [121].

There are also a number of studies that have begun to use pose estimation to measure *activity*-level behaviors. Gait assessment, in particular, has been an early clinical target for these evolving tools. Video-based tools have been used to successfully capture gait parameters such as step lengths, step width, step time, stride length, gait velocity, and cadence in people with stroke [122], PD [25,123] or dementia [124]. Beyond gait, the timed up and go is a widely accepted assessment of functional mobility in patients with a range of neurological disorders or disease. Li et al. recently validated and used a video-based activity classification to automatize timed-up-and-go sub-task segmentation (sit-to-stand, walk, turn, walk-back, sit-back) in people with PD [125].

Future work should focus on further validation of pose estimation with gold standard kinematic tools and interpretability alongside standard clinical assessments. Additional patient populations with a wide range of different movement patterns should be included in these investigations in order to develop algorithms that are broadly applicable. The potential of video-based analysis and pose estimation to quantitatively measure participation-level data in the home and the community should also be a top priority. Precise data captured in the real world not only will provide clinicians with important data from which they can make clinical decisions, but this may also facilitate early diagnosis of movement disorders and the ability to track movement patterns throughout a disease course.

We summarize many of the applications discussed in Section 4 in Table 1 below.

## 5. What Are the Limitations of Pose Estimation?

While many of our perspectives on the limitations of human pose estimation algorithms with regard to applications in human health and performance are embedded within the sections above, we considered that it may be helpful to include a condensed summary section here. As mentioned previously, technical limitations have been discussed extensively in prior reviews [20,21]. Here, we list perceived limitations in two general areas: *application limitations* and *barriers to implementation*. We consider application limitations to be those associated with obtaining high quality, usable data from video recordings via pose estimation (some are also discussed in [21]) and barriers to implementation to be limitations associated with the uptake and implementation of pose estimation approaches for common use among clinicians and researchers (with an emphasis on implementation in clinical settings).

### 5.1. Application Limitations

- Occlusions: these occur when one or more of the anatomical locations desired to be tracked are not visible. This may be due to occlusion by other body segments, by other people in the frame, or by inanimate objects (e.g., assistive devices—canes, walkers, crutches, orthoses, robotics; clinical objects—beds, hospital gowns, medical devices; sporting equipment—helmets, balls, bats, sticks).
- Limited training data: networks that are trained on sets of images that lack diversity (e.g., clothing, poses, illuminations, viewpoints, unusual postures associated with clinical conditions) may not perform well in applications where the videos are quite different from those included in the training set. Applications of current techniques that require a training dataset may require creation of a new training dataset if movements/images of a patient population are substantially different from those included in the existing training dataset (e.g., abnormal hand postures after stroke). This is particularly important given that most training datasets are biased toward healthy movement patterns.
- Capture errors: pose estimation algorithms may identify and track unwanted human or human-like figures in the field of view (e.g., people in the background, images on posters or artwork).
- Positional errors: tracking may be difficult when conditions introduce uncertainty into the positions of anatomical locations within the image (e.g., wearing a dress, hospital gown, athletic uniform or padding). This may also occur when attempting to track a movement from a suboptimal viewpoint (e.g., measuring knee flexion from a frontal view).
- Limitations of recording devices: use of devices with low sampling rates (e.g., the sampling rate of common video recording devices is often approximately 30 Hz) may be unable to capture accurate movement kinematics of movements that occur at high speeds or high frequencies. The aperture and shutter speed of recording devices can also impact image quality and introduce blurring, which can impact the quality of the tracking achieved through pose estimation.

**Figure 5:**
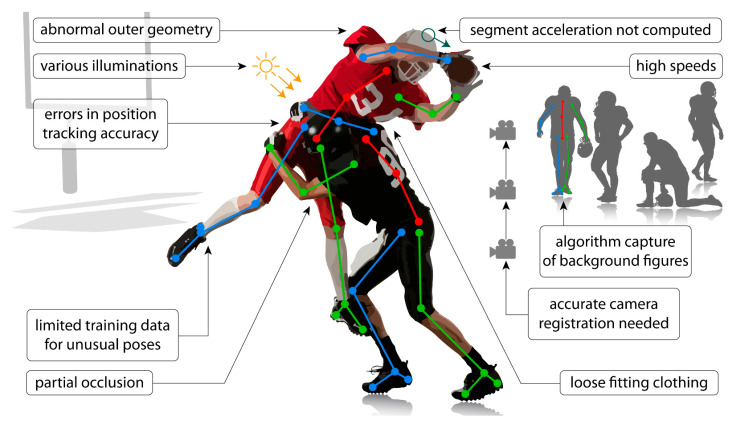
Common application limitations with current pose estimation algorithms and challenges with using these algorithms outside of the laboratory. These applications commonly require three-dimensional kinematics of multiple people moving at relatively high speeds to be tracked in environments with background figures (e.g., irrelevant people and objects shaped similarly to people). This leads to challenges with segment occlusion, unintentional capture of background figures, and registration of multiple cameras. Additionally, using current algorithms for scenarios different than the training dataset (e.g., different movements, different types of clothing or equipment being worn, different lighting) may lead to reduced accuracy in the predicted kinematics or, potentially, failure of the algorithm. Finally, most algorithms do not predict kinematic metrics that are required for some applications (e.g., head acceleration to assess concussion risk), and limitations with using current algorithms on time-series data make it challenging to accurately derive these metrics.

Examples of application limitations are depicted in Figure 5.

### 5.2. Barriers to Implementation

- User-friendliness: we currently lack plug-and-play options for pose estimation. While we certainly understand and acknowledge the many reasons for this, pose estimation is unlikely to be used widely in clinical settings in particular until user-friendliness improves. We outline several relevant components to user-friendliness below:
  ▪ Set up time: in our experience, many users want point-and-click capability. They want to be able to carry a recording device in their pocket, use it to record a quick video of their patient or research participant when needed, and ultimately obtain meaningful information about movement kinematics. Alternatively, they want a reserved space where a recording device could be permanently mounted and easily started and stopped (e.g., a tablet mounted to a wall). Any configuration that requires multi-camera calibration or prolonged set up time is unlikely to be adopted for widespread clinical use.
  ▪ Delayed results: many users want results in near real-time. There is a need for fast, automated approaches that immediately process the pose estimation outputs, calculate relevant movement parameters, and return interpretable data.
  ▪ Programming and training requirements: some existing pose estimation options are very easy to download, install, and use for users with basic technical expertise. However, even these can remain prohibitively daunting for clinicians and researchers without technical backgrounds. Technologies that require any amount of programming or significant training are unlikely to reach widespread use in clinical settings.
- Outcome measure challenges: in some cases, users want to use movement data to improve clinical or performance-related decision-making, but it is not immediately clear what parameters of the movement will lead to improved outcomes (e.g., a user may express interest in measuring “walking” but is not sure which specific gait parameters are most relevant to their research study or clinical intervention). Therefore, there is a desire to collect kinematic data, but how these data should be used is not well-defined. Similarly, in the case of clinical assessments, there needs to be a clear link to relevant clinical and translational outcomes—the users should have input as to what output metrics are important.
- Limited hardware infrastructure: as described above, some applications of pose estimation for human movement tracking require significant computational power. Some clinical and research settings are unlikely to have access to the hardware (e.g., GPUs) needed to execute their desired applications in a timely manner.
- Technology challenges: many technologies that promise potential for clinical or human performance impact are made available before they are fully developed. This can lead to buggy software and frequent updating, which harms trust and credibility among users. This can, in turn, exacerbate the hesitancy in adopting new technologies present in some clinical and research communities, especially in artificial intelligence technologies (such as pose estimation) that are purported to supplement or even replace expert human assessment.
- Lack of validation and feasibility data: there is a need for large-scale studies to validate pose estimation outputs against ground truth measures in a wide range of different populations. This may be accomplished in a variety of ways, including (but not limited to) comparisons with three-dimensional motion capture, wearable devices with proven accuracy, expert clinical ratings and/or assessments, or even possibly other pose estimation algorithms. The error (relative to the ground truth measurement) that is deemed acceptable is likely to depend on the use case and the metrics being used. In our experience, users who study very specific movements of joints or other anatomical landmarks (e.g., biomechanics or motor control researchers) are likely to seek greater accuracy than, for example, a clinician who may wish to incorporate a video-based assessment of walking speed as part of a larger clinical examination. It may be desirable to begin to develop field-specific accuracy standards for some applications.

There is also a need for testing of sensitivity, specificity, feasibility, and reliability. When a new clinical outcome measure is developed, a first step should be to establish criterion-validity or construct validity between the pose estimated measures and age-concurrent, clinician-coded, gold-standard clinical measures. Next, using receiver operating characteristic (ROC) analysis, sensitivity and specificity should be compared to assess the ability of the new pose estimated measure in predicting dichotomous outcomes (e.g., motor impaired vs. motor unimpaired). Area under the curve (AUC) should further be computed as a measure of the ability to distinguish between groups. Finally, it is important to evaluate the feasibility and acceptability of the new pose estimation protocol. One way to assess feasibility is to assess the number of completed and submitted usable videos by patients (i.e., the total number of videos submitted divided by the number expected, multiplied by 100). One way to assess acceptability is through satisfaction questionnaires/surveys. For example, after video submission, patients, families of patients (if patients are children), and clinicians can complete a brief satisfaction questionnaire/survey regarding their experience using the pose estimation protocol.

**Figure 6:**
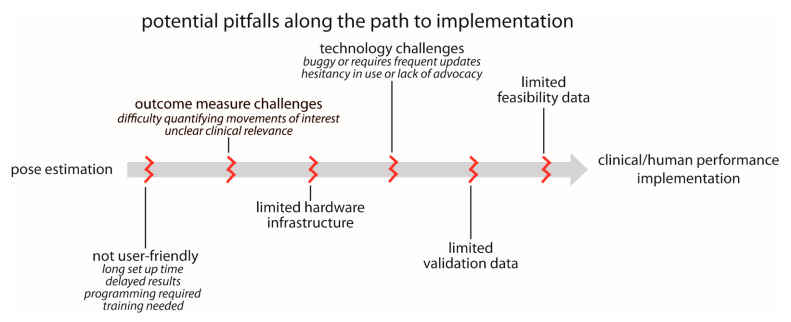
Common pitfalls that must be avoided on the path to widespread implementation of pose estimation applications for human health and performance. These are covered in greater detail in the “**What are limitations of pose estimation?**” section of the manuscript.

These potential pitfalls along the path to implementation are shown in Figure 6.

## 6. Conclusions

The emergence and continued development of human pose estimation approaches offer exciting potential for making quantitative assessments of human movement kinematics significantly more accessible. Pose estimation algorithms directly address an important and widespread need for low cost, easy to use, accessible technologies that enable human movement tracking in virtually any environment, including the home, clinic, classroom, playing field, and other ‘in the wild’ settings. Applications in health and human performance have begun to emerge in the literature, but we perceive that these technologies are still in their relative infancy with regard to the potential for research and clinical implementation. Many limitations persist, and it is important that users are aware of these and adjust expectations accordingly. However, we anticipate that applications of pose estimation in human health and performance will continue to expand in coming years, and these technologies will provide powerful tools for capturing meaningful aspects of human movement that have been difficult to capture with conventional techniques.

## Figures and Tables

**Table 1:** Summary of example applications of pose estimation in human health and performance across the lifespan.

Domain	Behavior/Movement Pattern Tracked	References
**Motor and non-motor development**	Infant cruising (early locomotion)	[36]
	Infant play/general movement	[37]
	Infant writhing	[51]
**Human performance optimization,** **injury prevention, and safety**	Healthy repetitive movements	[14]
	Healthy gait	[15,26,29,30,31,35,40]
	Sign language	[19]
	Healthy running	[27,35]
	Bilateral squat	[28]
	Healthy gait/jumping/throwing	[29]
	Lifting	[79,84]
	Various unsafe working behaviors	[80,81]
	ACL injury risk	[82,85,86]
	Handcart pushing and pulling	[83]
	Ergonomic postural assessment	[87]
	Remotely-delivered rehabilitation	[88,91,92,93]
	Healthy finger movements	[90]
	Rehabilitation robotics	[94,95,96,97]
	Athletic training	[100,101]
	Swimming	[102]
**Clinical motor assessment**	Gait in Parkinson’s disease	[25,33,123]
	Knee kinetics in osteoarthritis	[32]
	Gait in cerebral palsy	[34]
	Simulated abnormal gait	[72,74]
	Gait in older adults	[73]
	Fall detection	[76,77,78]
	Dyskinesias in Parkinson’s disease	[118,119,120]
	Gait in older adults with dementia	[124]
	Timed up-and-go in Parkinson’s disease	[125]

## Data Availability

Not applicable.
